# Intensity modulated radiotherapy for localized prostate cancer: rigid compliance to dose-volume constraints as a warranty of acceptable toxicity?

**DOI:** 10.1186/1748-717X-2-6

**Published:** 2007-01-15

**Authors:** Michael J Chen, Eduardo Weltman, Rodrigo M Hanriot, Fábio P Luz, Paulo J Cecílio, José C da Cruz, Frederico R Moreira, Adriana S Santos, Lidiane C Martins, Wladmir Nadalin

**Affiliations:** 1 Department of Radiation Oncology, Hospital Israelita Albert Einstein, Av. Albert Einstein, 627/701 – Sao Paulo, Brazil; 2 Department of Radiation Oncology, Hospital das Clínicas da Faculdade de Medicina da Universidade de São Paulo, Av. Dr. Enéas de Carvalho Aguiar, 255 – Sao Paulo, Brazil; 3 Instituto Israelita de Ensino e Pesquisa, Av. Albert Einstein, 627/701 – Sao Paulo, Brazil

## Abstract

**Background:**

To report the toxicity after intensity modulated radiotherapy (IMRT) for patients with localized prostate cancer, as a sole treatment or after radical prostatectomy.

**Methods:**

Between August 2001 and December 2003, 132 patients with prostate cancer were treated with IMRT and 125 were evaluable to acute and late toxicity analysis, after a minimum follow-up time of one year. Clinical and treatment data, including normal tissue dose-volume histogram (DVH) constraints, were reviewed. Gastro-intestinal (GI) and genito-urinary (GU) signs and symptoms were evaluated according to the Radiation Therapy Oncology Group (RTOG) toxicity scales. Median prescribed dose was 76 Gy. Median follow-up time was of 26.1 months.

**Results:**

From the 125 patients, 73 (58.4%) presented acute Grade 1 or Grade 2 GI and 97 (77.2%) presented acute Grade 1 or Grade 2 GU toxicity. Grade 3 GI acute toxicity occurred in only 2 patients (1.6%) and Grade 3 GU acute toxicity in only 3 patients (2.4%). Regarding Grade 1 and 2 late toxicity, 26 patients (20.8%) and 21 patients (16.8%) presented GI and GU toxicity, respectively. Grade 2 GI late toxicity occurred in 6 patients (4.8%) and Grade 2 GU late toxicity in 4 patients (3.2%). None patient presented any Grade 3 or higher late toxicity. Non-conformity to DVH constraints occurred in only 11.2% of treatment plans. On univariate analysis, no significant risk factor was identified for Grade 2 GI late toxicity, but mean dose delivered to the PTV was associated to higher Grade 2 GU late toxicity (p = 0.042).

**Conclusion:**

IMRT is a well tolerable technique for routine treatment of localized prostate cancer, with short and medium-term acceptable toxicity profiles. According to the data presented here, rigid compliance to DHV constraints might prevent higher incidences of normal tissue complication.

## Background

External-beam radiotherapy is the most utilized radiotherapy modality for treatment of localized prostate cancer and local control is related to delivered dose [1-4]. Three-dimensional conformal radiotherapy [3D-CRT) is a technique used to achieve this "dose escalation", but is limited by the consequent risk of excessive rectal and bladder complications [5,6].

Recently, the development of the intensity modulated radiotherapy (IMRT) has been shown to be a reasonable option to deliver higher radiation doses to prostate cancer patients, with acceptable low rates of complications [7-9].

This study presents a retrospective evaluation of the initial toxicity following the technical implementation of IMRT, for treatment of localized prostate cancer patients. Clinical and treatment related factors, including normal tissue dose-volume histogram (DVH) constraints, were analyzed as possible risk factors for gastro-intestinal (GI) or genito-urinary (GU) toxicity.

## Methods

### Selection of patients

Between August 2001 and December 2003, 132 consecutive patients with prostate cancer were treated with IMRT. From this patient group, 125 patients with a minimum follow-up time of one year were considered evaluable to acute and late toxicity analysis, as they were staged as with localized disease and treated with IMRT as a sole treatment, or in adjuvant manner, after surgical resection, to the prostatic bed. Data regarding patient clinical and staging characteristics are shown on Table 1.

**Table 1:** Patients characteristics

	Patients number
Age:	
≤ 65	43 (34.4%)
> 65 e ≤ 75	57 (45.6%)
> 75	25 (20.0%)
Highest serum PSA level (ng/ml):	
≤ 10	77 (61.6%)
> 10 e < 20	31 (24.8%)
≥20	17 (13.6%)
Stage (AJCC 1997):	
T2aN0M0 or lower	88 (70.4%)
T2bN0M0 or higher	37 (29.6%)
Gleason Score:	
≤ 6	63 (50.4%)
7	49 (39.2%)
8 – 10	13 (10.4%)
Exclusive radiotherapy treatment	90 (72.0%)
Post-operative radiotherapy:	
adjuvant treatment	16 (12.8)
PSA-relapse rescue	19 (15.2%)
Neo-adjuvant hormonal therapy	57 (45.6%)

At admission, all patients had a positive histologic diagnosis of prostate cancer, graded according to Gleason Score specification [10]. The 1997 American Joint Commission on Cancer (AJCC) staging system [11] was utilized and, specifically for operated patients, surgical staging was done based on anatomic-pathological information. Also, patients were stratified into prognostic groups, according to criteria adapted from the data published by Bolla *et al. *[12]. This stratification was also used as an "in house" treatment guideline to IMRT dose levels prescription (Table 2).

**Table 2:** Prognostic groups stratification and radiation doses prescriptions:

	Prognostic groups^§^		
	Low Risk	Intermediate Risk	High Risk

Highest serum PSA level (ng/ml)	≤ 10 AND	> 10 e < 20 OR	≥ 20 OR
Stage (AJCC 1997)	≤ T2aN0M0 AND	T2bN0M0 OR	≥ T3N0M0 OR
Gleason Score	≤ 6 (3 + 3)	7	≥ 7 OR 2 Intermediate Risk factors associated
Patients number*	38 (30.4%)	28 (22.4%)	57 (45.6%)
Neo-adjuvant hormonal therapy	12/38	14/28	31/57
Post-operative therapy	6/38	4/28	25/57

^§ ^Suggested radiation dose prescription: 72 Gy to post-operative radiotherapy, 74 Gy to low risk, 76 Gy to intermediate risk and 78 Gy to high risk patients, respectively PTV: planning target volume

* 2 patients could not have their risks assessed

There was no restriction concerning hormone therapy and the usage was determined by physician's discretion, as an adjunct treatment to reduce prostatic volume or to "high risk" patients.

### Radiotherapy planning

At the moment of the IMRT technique implantation, a class solution was established to be applied to all treatment plans. Before effective IMRT delivery, all patients were submitted to a pelvic CT simulation (CT-Sim) procedure. Using the CT-Sim data, and for planning calculation, the following structures were contoured: femoral heads, prostate and seminal vesicles, bladder and rectum (entirely contoured from the anal canal to rectum-sigmoid transition). The clinical target volume (CTV) corresponded to prostate and the entire seminal vesicles. For operated patients, (i.e.: after radical prostatectomy), the CTV corresponded to the prostatic and seminal vesicles bed, according to pre-operative CT or MRI scans. Margins of 0.6 cm (posterior) and 1.0 cm (cranial, caudal, anterior and laterals) were applied to the CTV when defining the planning target volume (PTV).

All patients were treated at a Clinac 23-EX^® ^linear accelerator (Varian Medical Systems, Palo Alto, CA, USA), utilizing a dynamic IMRT technique ("sliding window"), with a 5 isocentric coplanar beam arrangement and photons with beam energy of 15 MVs. Inverse planning was calculated using the Helios^® ^software (Varian Medical Systems, Palo Alto, CA, USA), according to pre-established DHV constraints and treatment dose specifications (Table 3, based on previously published data [5-7,13]). Daily prescribed dose was of 200 cGy.

**Table 3:** Dose-volume histogram and treatment volumes constraints:

Structure	Maximum Volume/Maximum Total Dose		
Bladder	≤ 55%/≥ 47 Gy	≤ 30%/≥ 70 Gy	Maximum dose: 82 Gy
Rectum	≤ 55%/≥ 47 Gy	≤ 40%/≥ 65 Gy	≤ 25%/≥ 70 Gy
	≤ 10%/≥ 75 Gy	Maximum dose: 82 Gy	
Femoral head	Maximum dose: 50 Gy		
PTV	Maximum dose ≤ 20% of prescription dose to PTV Minimum dose of 70 Gy if prescription dose of 72 Gy to PTV Minimum dose of 72 Gy if prescription dose of 74 Gy to PTV Minimum dose of 74 Gy if prescription dose of 76 Gy to PTV Minimum dose of 76 Gy if prescription dose of 78 Gy to PTV PTV's coverage to a minimum of 95% of the entire volume		

PTV: planning target volume

Immobilization and target localization's verification were regularly done utilizing a customized anatomical pelvic mold and weekly isocenter's anterior-posterior and laterals radiographs. Furthermore, all patients were ordered to evacuate before and keep the bladder full during the CT-Sim and all the daily applications, according to a proper routine. Treatment started effectively only after plan approval by both the radiation oncologist and the medical physicist and after "quality assurance" testing, also according to a proper routine.

All IMRT treatments were delivered successfully and median follow-up time was 26.1 months (range: 12.1 to 42.2 months). The median prescribed dose was 76 Gy, (range: 68 to 78 Gy), and the mean administered dose was 76.5 Gy, with median maximum and minimum doses of 81.8 Gy and 72 Gy, respectively.

### Toxicity evaluation and follow-up

Data collection was done by retrospective review of medical files. Also, for each patient, a GI and GU toxicity assessment profile was created by the time of the IMRT treatment. Data obtained included relevant previous medical history (i.e.: diabetes, hypertension, previous surgery and ano-rectal or urinary diseases), medications and GI and GU symptoms. During treatment, all patients were evaluated on a weekly basis, regarding any new or worsening symptoms. Afterwards, patients were suggested to return to consultation with the radiation oncologist regularly, for clinical and digital rectal evaluation, which also included appraisal of GI and GU symptoms and serum PSA levels.

Acute toxicity was defined as the appearance or worsening of any GI or GU symptoms during treatment time or until after 6 months of it. Late toxicity was defined the same way, but after the 6th month of follow-up time. Either acute or late toxicity grading was scored based on the respective toxicity scales proposed by the Radiation Therapy Oncology Group (RTOG) [14-16].

### Statistical analysis

Univariate exact logistic regression [17] was applied to test the association between any potential predictor and RTOG Grade 2 toxicity or higher. All significance probabilities (p values) presented are two-sided and values lower than 0,05 were considered statistically significant. "Odds ratios" and their respective 95% confidence intervals were estimated. The Logxact 6.3^® ^software (Cytel Software Corporation, Cambridge, MA, USA) was utilized in all the statistical analysis.

## Results

Of all the patients, 60.0% (75 patients) and 80.0% (100 patients) presented some grade of acute GI or GU toxicity, respectively. Acute Grade 3 GI toxicity occurred in 2 patients (1.6%, a case of diarrhea requiring parenteral support and a case of severe blood discharge necessitating sanitary pads), and acute Grade 3 GU toxicity occurred in 3 patients (2.4%, all of them with frequency of urination or nocturia of more than every hour, with urgency, dysuria and irritative symptoms). For late toxicity, of all the patients, 20.8% (26 patients) presented some grade of late GI toxicity and 16.8% (21 patients) presented some grade of GU toxicity (Table 4). Late Grade 2 GI toxicity occurred in 6 patients (4.8%, a case of episodes of moderate diarrhea but frequently requiring parenteral support, two cases of frequent episodes of moderate diarrhea and colic and three cases of frequent episodes of intermittent bleeding, but requiring minor non-surgical procedures). Late Grade 2 GU toxicity occurred in 4 patients (3.2%, all of them with moderate frequency with urgency, dysuria and irritative symptoms).

**Table 4:** Acute and late gastro-intestinal (GI) and genito-urinary (GU) toxicities profiles:

	Pre-treatment		Acute		Late	
	GI	GU	GI	GU	GI	GU

RTOG G1	3 (2.4 %)	19 (15.2%)	54 (43.2%)	47 (37.6%)	20 (16.0%)	17 (13.6%)
RTOG G2	None	2 (1.6%)	19 (15.2%)	50 (40.0%)	6 (4.8%)	4 (3.2%)
RTOG G3	None	None	2 (1.6%)	3 (2.4%)	None	None
RTOG G4/G5	None	None	None	None	None	None

RTOG: Radiation Therapy Oncology Group

Treatment plans were able to be performed with a high level of compliance to DVH constraints and for only 14 of the 125 patients (11.2% of the cases) there was some degree of non-conformity, with doses 3% higher than the acceptable value for each constraint. Regarding this, violations were more frequent when patients were prescribed to lower dose levels, with 7 cases (16.1%) out of the 44 patients receiving prescription doses of 74 Gy, and at "inferior" DVH constraints' levels (lower dose levels and bigger volumes). On the contrary, violations were rare for the "superior" DVH constraints' levels (higher dose levels and smaller volumes) and, for each organ (i.e.: bladder and rectum) and constraint, level of compliance was above 90% (Table 5).

**Table 5:** Compliance to DVH constraints as to different prescription dose:

	Prescription doses levels					
	D74 (n = 44 patients)		D76 (n = 40 patients)		D78 (n = 41 patients)	

	Rectum	Bladder	Rectum	Bladder	Rectum	Bladder

D55 (Gy)	93.2% (41/44)	93.2% (41/44)	90% (36/40)	100%	95.1% (39/41)	100%
D30 (Gy)	Not evaluated	100%	Not evaluated	100%	Not evaluated	100%
D25 (Gy)	97.7% (43/44)	Not evaluated	100%	Not evaluated	100%	Not evaluated
D10 (Gy)	100%	Not evaluated	100%	Not evaluated	100%	Not evaluated
Dmax (Gy)	100%	100%	97.5% (39/40)	100%	100%	100%

D74: doses up to 74 Gy; D76: doses from 74 to 76 Gy; D78: doses of 78 Gy; D55: dose at 55% of the volume (rectum or bladder); D30: dose at 30% of the volume (rectum or bladder); D25: dose at 25% of the volume (rectum or bladder); D10: dose at 10% of the volume (rectum or bladder); Dmax: maximum dose (rectum or bladder)

By performing an evaluation of possible factors related to acute toxicity, it was observed that a patient's personal history of systemic arterial hypertension was a significant risk factor for Grade 2 or higher GI acute toxicity (p = 0.042). However, for Grade 2 or higher GU acute toxicity, significant risk factors were both minimum and mean PTV doses (p = 0.049 and 0.042, respectively), and also the patient's surgical "status" when treated with RT, (if previously operated or not, p = 0.009) (Table 6).

**Table 6:** Univariate analysis of prognostic factors to grade 2 or higher acute and late gastro-intestinal (GI) and genito-urinary (GU) toxicities:

	*P *values			
Variable	acute GI	acute GU	late GI	late GU

Surgical status: operated	0.667	0.009	1.000	0.527
Neo-adjuvant hormonal therapy	0.939	0.115	0.137	0.492
Diabetes mellitus	0.428	0.889	0.317	0.825
Systemic Arterial Hypertension	0.042	0.905	1.000	1.000
Dmean (Gy)	0.176	0.042	0.600	0.042
Dmax (Gy)	0.118	0.215	0.702	0.202
Dmin (Gy)	0.178	0.049	0.205	0.582
D55 (Gy)	0.164	0.885	0.568	0.552
D30 (Gy)	--	0.593	--	0.860
D25 (Gy)	0.889	--	0.426	--
D10 (Gy)	0.352	--	0.321	--
V47 (%)	0.088	0.614	0.583	0.626
V70 (%)	0.731	0.394	0.852	0.696

D55: dose at 55% of the volume (rectum or bladder); D30: dose at 30% of the volume (rectum or bladder); D25: dose at 25% of the volume (rectum or bladder); D10: dose at 10% of the volume (rectum or bladder); Dmean: mean dose to PTV; Dmax: maximum dose to PTV; Dmin: minimum dose to PTV; V47: volume receiving 47 Gy (rectum or bladder); V70: volume receiving 70 Gy (rectum or bladder)

No significant risk factor for GI Grade 2 or higher late toxicity was observed. Mean PTV doses correlated to GU Grade 2 or higher late toxicity as a significant risk factor (p = 0.042).

Results of local control, disease-free survival, PSA relapse-free survival or even global survival were not assessed, at the present study.

## Discussion

IMRT raised to radiation oncologists a possibility of tumour dose escalation without compromising doses to normal tissues. Since an initial publication by Zelefsky *et al. *[9], clinical utilization of this technique has been demonstrated to be safe, with acute and late rectal and bladder complications incidences at "acceptable" levels [8,18-21]. This report adds some information about routine usage of dynamic IMRT technique, describing treatment complication frequencies, in a small, but consecutive sample of localized prostate cancer patients. Results of late GI and GU toxicities of about 15% (Grade 1) and lower than 5% (Grade 2) published here are very comparable to what has already been shown elsewhere [19,21], as well as the elevated frequency of acute GI and GU toxicities, beyond 50%, but with rare cases of more severe complications [18-20].

Although results might seem to be very similar, proposed criteria to toxicity evaluation are very heterogeneous among the different already published reports. This analysis, however, was conceived taking advantage of widely used and very simple tools, which are the RTOG toxicities scales, in order to make data here easily understandable. An example is the report from Zelefsky *et al. *in which a higher importance was given to rectal bleeding as a sign of increased toxicity. Some of the bleeding complications were scored as Grade 3, (0.5% versus 1.5% for Grade 2 symptoms frequency), due to necessity of transfusion or laser cauterization procedures [19]. In the data demonstrated here, however, of the total number of six patients (4.8%) who presented GI Grade 2 late toxicity, only three of them (2.4%) presented rectal bleeding which necessitated laser cauterization procedures and all of them remitted after treatment, neither evolving to obstruction nor to bleeding requiring surgery (data not shown).

On univariate analysis it was not observed any significant association between clinical factors or DVH constraints and risk of GI late toxicity, which could predict a Grade 2 or higher index, as is usually described [5-7,22-24]. Frequency of complications was certainly low enough and there is no point to draw any precipitated conclusions about predisposing factors to rectal and bladder toxicity. A criticism to the data presented here could obviously be the negative influence of a heterogeneous group and of different treatment doses. These facts has certainly underpowered the analysis, leading to the absence of more instigating results.

Nonetheless, it must be stressed the lack of any Grade 3 late toxicity during the follow-up time, and an important reason might have been the rigid compliance to DVH constraints. Although the limit of acceptance for compliance to the DHV constraint levels was a random value of 3%, there was, as previously shown, a low rate of non-conformity to the them and, for the 14 patients with some degree of non-conformity, the "violation severity" was also of less than 6.5% (mean value, range: 3% – 21.3%).

As described earlier, the constraints utilized in the present study were elaborated based on data previously published in the literature. At the present moment, there is no ideal "set" of DVH constraints to be safely used, although there are some of these parameters that seem to be very strong predictors of GI and GU toxicity [22]. In this sample, the "set" of DVH constraints presented seemed to be reliable, as the preliminary toxicity results were very acceptable.

## Conclusion

Intensity modulated radiotherapy is a tolerable treatment technique for localized prostate cancer. Care must be taken, however, when applying literature data to daily practice, especially concerning dose escalation and the ensuing risks of normal tissue complications. A rigid compliance to dose-volume constraints derived from previously published experiences must always be observed as an additional tool to reduce treatment related risks and might be warranty of acceptable toxicity.

## Competing interests

The author(s) declare that they have no competing interests.

## Authors' contributions

MJC carried out the data collection and drafted the manuscript. EW conceived and coordinated the study. RMH, FPL and WN provided comments, critique and suggestions for its improvement. PJC and JCC carried out radiotherapy planning and provided comments, critique and suggestions for its improvement. FRM performed the statistical analysis. ASS and LCM participated in the data collection and in radiotherapy planning. All authors read and approved the final manuscript.
